# Smartpath^k^: a platform for teaching glomerulopathies using machine learning

**DOI:** 10.1186/s12909-021-02680-1

**Published:** 2021-04-29

**Authors:** Nayze Lucena Sangreman Aldeman, Keylla Maria de Sá Urtiga Aita, Vinícius Ponte Machado, Luiz Claudio Demes da Mata Sousa, Antonio Gilberto Borges Coelho, Adalberto Socorro da Silva, Ana Paula da Silva Mendes, Francisco Jair de Oliveira Neres, Semíramis Jamil Hadad do Monte

**Affiliations:** 1 Department of Specialized Medicine, Federal University of Piauí, Teresina, PI Brazil; 2 Open and distance education center and computer scientist of the Immunogenetics and Molecular Biology Laboratory (LIB - UFPI), Federal University of Piauí, Teresina, PI Brazil; 3 Department of Computing and computer scientist of the Immunogenetics and Molecular Biology Laboratory (LIB - UFPI), Federal University of Piauí, Teresina, PI Brazil; 4 Systems analyst at the Immunogenetics and Molecular Biology Laboratory, Federal University of Piauí, Teresina, PI Brazil; 5 Department of Biology and vice coordinator of the Immunogenetics and Molecular Biology Laboratory (LIB - UFPI), Federal University of Piauí, Teresina, PI Brazil; 6 Student of the Computing course at Federal University of Piauí, Teresina, PI Brazil; 7 Department of General Clinic and coordinator of the Immunogenetics and Molecular Biology Laboratory (LIB - UFPI), Federal University of Piauí, Teresina, PI Brazil

**Keywords:** Intelligent system, Renal pathology, Machine learning, Digital pathology, Kidney

## Abstract

**Background:**

With the emergence of the new coronavirus pandemic (COVID-19), distance learning, especially that mediated by information and digital communication technologies, has been adopted in all areas of knowledge and at all levels, including medical education. Imminently practical areas, such as pathology, have made traditional teaching based on conventional microscopy more flexible through the synergies of computational tools and image digitization, not only to improve teaching-learning but also to offer alternatives to repetitive and exhaustive histopathological analyzes. In this context, machine learning algorithms capable of recognizing histological patterns in kidney biopsy slides have been developed and validated with a view to building computational models capable of accurately identifying renal pathologies. In practice, the use of such algorithms can contribute to the universalization of teaching, allowing quality training even in regions where there is a lack of good nephropathologists. The purpose of this work is to describe and test the functionality of SmartPath^k^, a tool to support teaching of glomerulopathies using machine learning. The training for knowledge acquisition was performed automatically by machine learning methods using the J48 algorithm to create a computational model of an appropriate decision tree.

**Results:**

An intelligent system, SmartPath^k^, was developed as a complementary remote tool in the teaching-learning process for pathology teachers and their students (undergraduate and graduate students), showing 89,47% accuracy using machine learning algorithms based on decision trees.

**Conclusion:**

This artificial intelligence system can assist in teaching renal pathology to increase the training capacity of new medical professionals in this area.

**Supplementary Information:**

The online version contains supplementary material available at 10.1186/s12909-021-02680-1.

## Background

The Covid-19 pandemic has made education at all levels a major challenge around the world. Universities have been forced to implement changes in their traditional teaching methods based on blackboards and textbooks [1], and in the specific field of biomedical sciences, educators have used e-learning platforms to materialize complex biological concepts while overcoming unprecedented geographical barriers [2]. E-learning has shown promise not only in the economically viable integration of the teaching-learning systems [3], but it has also allowed learning at any time and place without needing the presence of an instructor [4]. The validity of the application of this powerful teaching tool is shown by reports of the superior performance of students who use them as an auxiliary tool in the educational process to that of students in the traditional classroom [5].

E-learning platforms, as well as virtual laboratories, have an enormous potential to catalyze the learning of complex disciplines and constitute a niche to be explored in teaching the diagnosis of pathologies. In fact, pathologies such as renal diseases, of which about 80% are glomerular in nature and require renal biopsy and analysis for diagnosis and therapeutic management, can benefit greatly from this strategy. This is because although renal biopsy is an old and low-risk procedure, it requires the presence of a pathologist with specialized training in nephrolopathology and a complex laboratory structure in terms of microscopy, which enhances the differences in access to professional training. The formation of the nephropathologist takes time, requiring extensive analytical training due to the structural richness of the glomerulus and the glomerular filtration barrier, a complex structure formed by three layers—the vascular endothelium, the glomerular basement membrane, and the podocytes with their spaced podocyte processes (pedicels) that need to be carefully evaluated. In addition, it requires the analysis of extra glomerular structures such as renal vessels, interstices, and tubules, as well as access to clinical and laboratory data for the pathological diagnosis of glomerular disease [6–8].

Because of all this complexity, strategies have been sought in the digital age to replace the repetitive and exhaustive tasks of the histopathological analysis of the nephropathologist in conventional microscopy with machine learning algorithms [9]. In this direction, the study of pathologies in digital form has been adopted as a new analysis strategy using virtual microscopy, with slides of conventional histological preparations with special stains for optical microscopy and immunofluorescence slides scanned entirely in specialized equipment and displayed on the screens of computers [10–12].

The advantages of teaching using digital pathology are clear, for in addition to making good a lack of laboratory facilities, it affords students appropriate experience at low cost and provides self-learning strategies. In addition, due to its digital nature, a virtually unlimited number of students can be taught simultaneously without the need for physical handling of histological pieces, which minimizes biological risks and ethical complications. With the perfect triangulation between digitization of slides (which produces images of complete slides), high-resolution digital cameras, and modern bioinformatics resources, it is possible to develop computational tools that can access this digital data and manipulate it for different purposes [13–17].

A bioinformatics resource increasingly used in academic and diagnostic medicine is machine learning which allows the development of tools capable of recognizing patterns and using them in the construction of computational models capable of solving problems [18, 19]. In machine learning (ML), after the training process, the machine itself finds the hypothesis that will best perform the diagnoses, considering the cases already analyzed [20].

Thus, automated processing to support the analysis of renal biopsy can improve the teaching and effectiveness of renal pathology, contributing to a more objective and standardized diagnosis, especially in regions where there is a lack of nephropathologists. This can be confirmed by the increase in the number of machine learning applications in renal pathology in recent years [7]. Initial studies using AI in nephropathology validated the method’s accuracy in identifying the standard histological lesions common in glomerular diseases, prompting researchers to continue using AI concepts and tools to complement the arsenal for nephropathology diagnosis [9, 21], however there is no computer program for universalizing analysis or for teaching nephropathology, yet. As ML applications in renal pathology for educational purposes and medical training are in an incipient stage, so to introduce digital pathology into the classroom, we sought to determine whether ML algorithms have acceptable accuracy in relation to conventional microscopy for the development of a tool that facilitates educational training in the teaching of glomerulopathies.

Thus, this study aims to describe and test the functionality of SmartPath^k^, a tool using machine learning to support teaching in glomerulopathies. For this purpose, we use an algorithm called J48, which creates a computational model of the respective decision tree.

J48 uses a divide-to-conquer approach to assemble the tree, whereby a complex problem is decomposed into simpler subproblems, recursively applying the same strategy to each subproblem, dividing the space defined by the attributes into subspaces, and associating them with a class [19]. The algorithm interrupts the data segmentation process once a level of similarity between the final classes previously determined by the user is reached. These data subdivisions are based on statistical procedures that consider the errors of the nodes and their descendants. The identification of the root and its descendants is given through calculations of entropy and the gain ratio of the information. Entropy is used to assess the predictive ability of an attribute, while the gain ratio is used at each node to choose the attribute that most efficiently subdivides the sample set into homogeneous subsets and characterizes the classes [18].

The decision tree model created by J48 will serve as a guide for creating quizzes to assess the SmartPath^k^ user’s learning. Once the model has been trained through cases previuously analyzed by the nephropathologist, the knowledge of the diagnoses is reflected in the system and, as new cases are added, the training of the model is redone and this knowledge is updated.

## Methods

In this study, we used supervised machine learning to induce a classifier (or hypothesis), using a previously labeled data set (glomerulopathies) to classify new unlabeled cases. This classifier model will be based on a decision tree. Decision trees are constituted by a hierarchical structure that acts as a means of forecasting / classification [22]. At all levels of the tree, decisions are made about the structure of the next level, until the end nodes are reached. This method is supervised, where the response variable is explained at the expense of the independent variables measured on any scale. Despite being used for prediction, the decision tree model will induce the construction of SmartPath^k^ quizzes. Therefore, the intelligence of the system comes from the dynamic creation of the steps in the quizzes (path from the tree to the diagnosis).

This study was carried out in five phases performed in creating the predictive model, namely, (1) data collection, (2) data preparation, (3) model training, (4) model validation, and (5) development of the software.

### Data collection

The data were obtained from the review of all histological reports of renal biopsies performed at the LAPAC Surgical Pathology Laboratory from 2013 to 2018. For the preparation of each renal biopsy report, 04 or 05 slides with different stains were analyzed (Hematoxylin-eosin, PAS, Masson’s Trichrome, Jones Silver, and Congo red) and in different magnifying objectives (40x, 100x, 400x, and 1000x). In all, 584 reports were collected and analyzed manually by the researcher pathologist to select those that contained the following information regarding the case: clinical data, laboratory data, and the diagnostic hypothesis of kidney disease, histological biopsy study (light microscopy, special staining and at least immunofluorescence study), and conclusive histological report of a nosological entity of glomerular disease. From these, 100 reports were separated for the study, which included 30 nosological entities of glomerular diseases in the pathology (see Additional file 1) analyzed in slides with the different special stains used in the routine (Hematoxylin-eosin, silver methenamine, Schiffer’s acid, and Masson’s Trichrome).

Initially, the information contained in the reports was translated into a spreadsheet. Each column of the spreadsheet is now called an attribute, and each row an instance, representing a report. The cells in this spreadsheet received categorical information present or absent for some attributes, and others received specifications according to each attribute evaluation. The product of this phase was a table with categorical variables and with the histological diagnosis defined and reviewed by the researcher.

Next, images were separated and revised from these 100 biopsy reports representative of the 30 diagnosed nosological entities used as a static image for the construction of the Teaching computer program called SmartPath^k^.

### Data preparation

In this step, the data received in a spreadsheet were reviewed for transformation into a format compatible with the input required by the J48 algorithm. For this cleaning, duplications and redundancies, as well as spelling errors, were manually removed.

### Model training

**Fig. 1:**
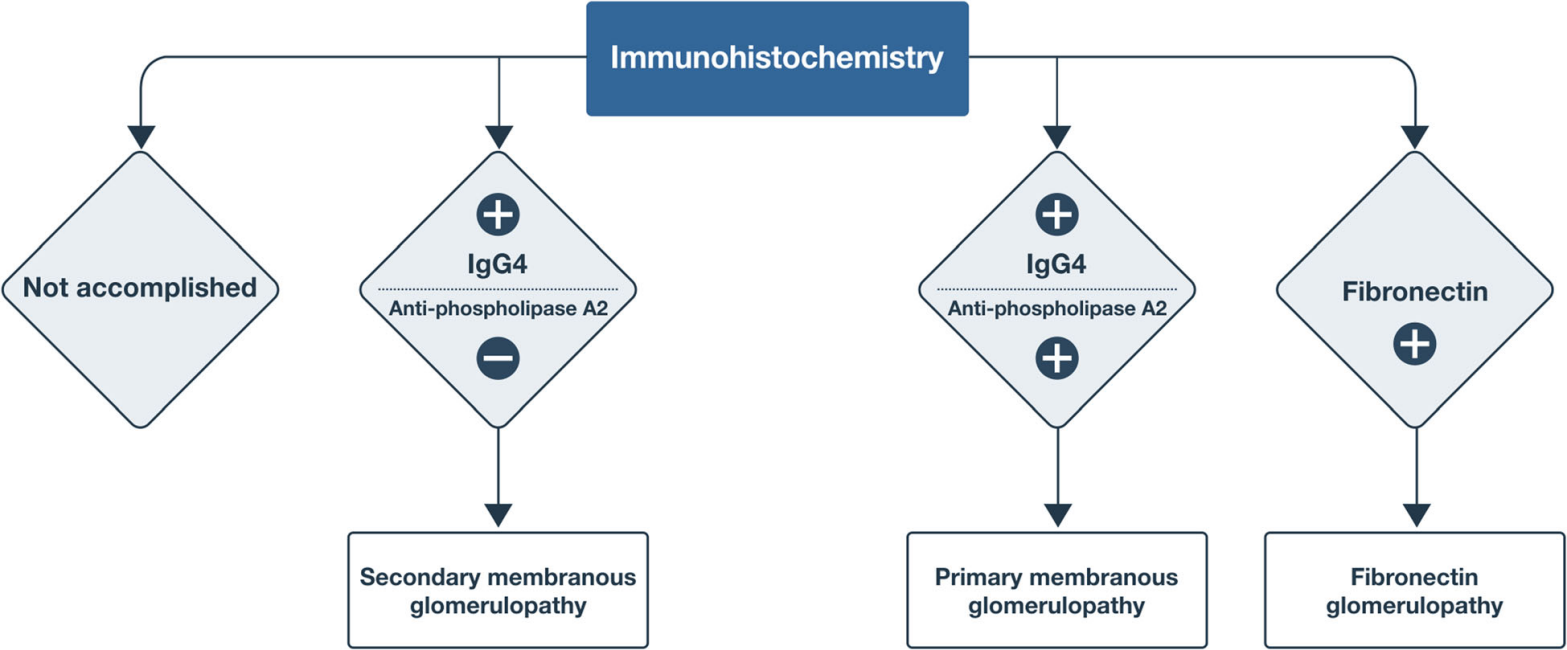
Schematic design of one of the branches of the decision tree

For this training, 80 instances were randomly selected with their respective diagnoses and associated images, keeping 20 instances for the validation step. The revised file was entered as input to the J48 algorithm. The product of this stage is a decision tree trained to infer the histological diagnosis of the case. Figure 1 illustrates a segment of the tree resulting from the J48 algorithm for the database of this research. In this case, we can observe that the immunohistochemistry attribute will be evaluated. Depending on the value of the attribute, a diagnosis might be reached or might not without accessing another attribute.

For example, if the immunohistochemistry value is equal to “positive for fibronectin,” the diagnosis of the evaluated case will be glomerulopathy for fibronectin and will not need another attribute to infer the diagnosis of glomerular disease.

### Model validation

Several aspects can be used to evaluate a model, such as the accuracy of the model generated, the comprehensibility of the extracted knowledge, the learning time, and the storage requirements of the model. The model used in this study is predictive; therefore, we evaluate it with measures related to its performance on the predictions it makes.

The results of the model validation were obtained through the Cross Validation method, which divides the set of data into K partitions (Folds) and then randomly separates one part for testing and conducts the training with the others. This procedure is repeated for all parts and at last the accuracy average among the parts is calculated. In our case, we used 10 folds always with the 80 samples or training instances and the 20 instances not used to train the model served to evaluate the learning of the system in correctly classifying the tested diagnosis. Thus the accuracy was 89.47% with a value of kappa of 0.88, which is characterized as adequate for correctly predicting the diagnosis, thus validating the training of the model. In this way, a trained model was obtained, potentially useful for the development of a computer program to support teaching in glomerulopathies using the decision tree generated by J48.

We note that the separation of the data into 80 cases for training and 20 for tests served only to properly evaluate the performance of the model. In fact, the model will be generated by training on all 100 examples, enhancing its accuracy.

### Software development

#### Internal architecture

Based on the trained predictive model, we developed the system’s user interface. SmartPath^k^ is a web application developed in the Python language using the Django framework. SmartPath^k^ can be accessed from different platforms, e.g., PCs, tablets, and smartphones, because it works entirely in a Web environment. The Bootstrap framework was used to support its layered model-view-controller architecture. These technologies were chosen because they are open-source and are secure, robust, and easy to learn and use. The system is available in Portuguese and English.

The SmartPath^k^ integrates, in a way transparent for the end user, the training decision tree responsible for the automatic generation of the quizzes and the selection of the static image of the biopsy blade representative of the diagnosis inferred by the tree. Therefore, the images are associated with each diagnosis and will be presented to the user with the quiz for each case. The quiz in turn represents the best path taken by the tree to reach the diagnosis.

The decision tree model is the intelligent part of the system because it reflects the cases diagnosed by the pathologist in an autonomous and current way. In addition, it automatically indicates the main aspects (attributes) that were considered for the result, allowing the student to explore each of the variables that will lead him to determine the final diagnosis and thus test the studied content.

The decision tree is updated dynamically through the module for inserting new cases. This system module was developed so that the administrator himself or a designated user proceeds with the system update through a single SmartPath^k^ interface. When new cases are inserted, SmartPath^k^ generates a new decision tree and a new accuracy. It is also noteworthy that the entry of new cases will cause a change in the tree structure and, theoretically, a change in the knowledge represented by the tree. That is, when new cases are incorporated into the system, the system’s capacity to teach is increased.

## Results

### Using the tool

SmartPath^k^ was developed as a complementary remote tool in the teaching-learning process for pathology teachers and their students (undergraduate and graduate students). In order to obtain better results with tool, users must have prior knowledge of the histology of the renal glomerulus and the most prevalent glomerulopathies, therefore requiring histology as a minimum.

The system has four user profiles: Administrator, Teacher, Pathologist, and Student. The Administrator can perform any action on the System. The teacher manages the classes, so he has access to the actions associated with class and student management. As in a virtual learning environment, the teacher can create, edit, and delete a class, as well as enroll and remove students from the class. The Pathologist has the same permissions as the teacher and, in addition, can add, remove, and edit cases. It is up to the Student to solve the quizzes to measure the knowledge acquired through the available levels of difficulty.

Focusing on ease of learning and use, the user interface is intuitive and implements simple and agile navigability through a flow that translates into few interaction screens. In this way, the system provides a simplified usage experience where the student can observe the changes in the image, answer the questionnaires, and verify their performance in solving them.

#### Student interaction flow

**Fig. 2:**
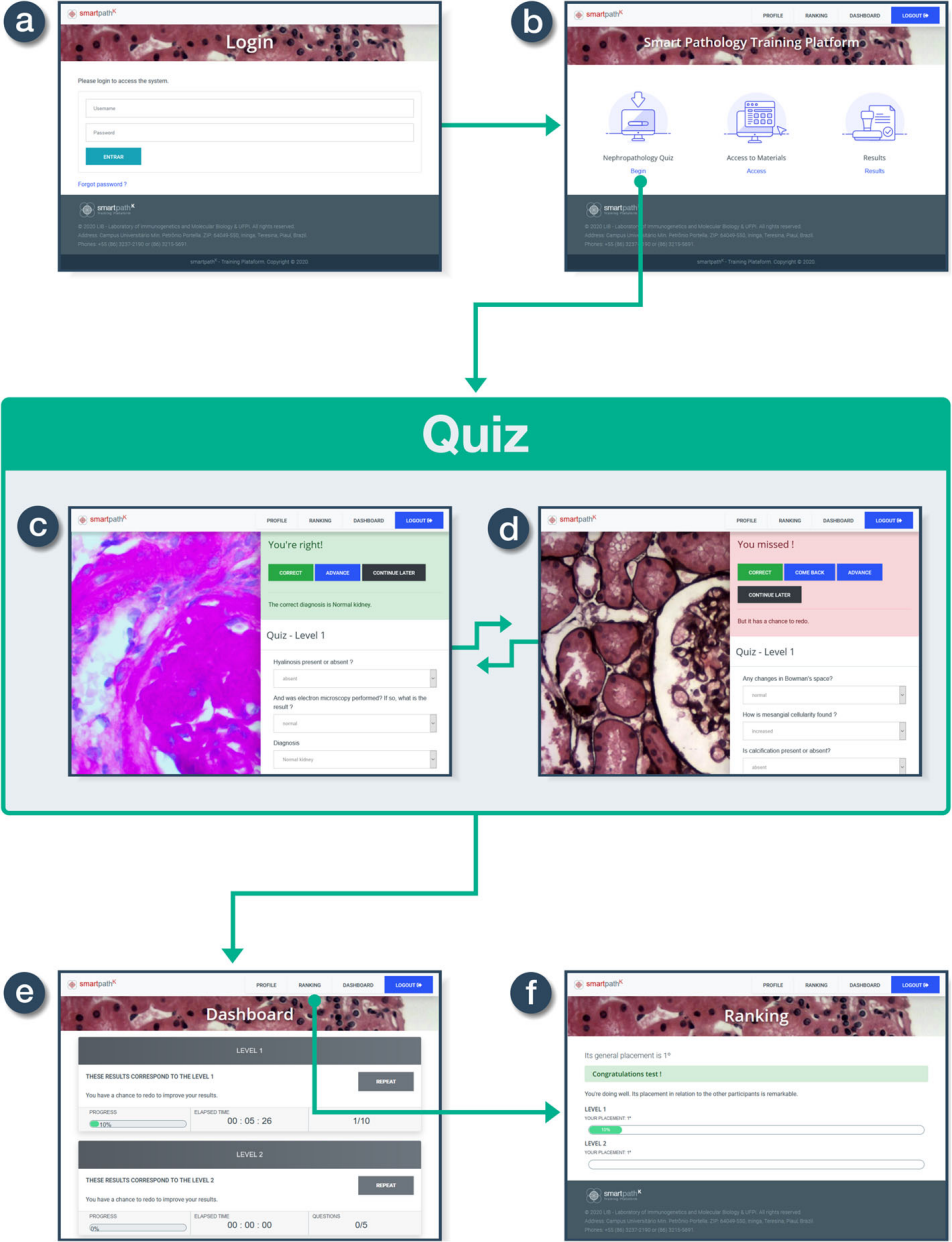
Infographic - Student interaction flow. Screen **a**: Login; Screen **b**: Quiz access; Screen **c** and **d**: Quiz correct answers and errors; Screen **e**: Dashboard; Screen **f**: Ranking

After logging into the system (Fig. 2a), the users will be directed to the home page, where they can access the questionnaire, supplementary materials, and results (Fig. 2b). To access the questionnaires, they click on “Start” and the system will randomly present a questionnaire to be answered. The questionnaires will have different numbers of questions, according to the decision tree. The tree built by the machine uses the fewest attributes possible to arrive at the correct answer (diagnosis). In this case, the questions will flow in the direction of the tree generated by the J48 algorithm from top to bottom. After choosing the answers for each field, the questionnaire will be corrected (Fig. 2c). If it is correct, a message will be displayed and a new questionnaire will then be presented. If the answers given by the student do not correspond to the flow of the tree, the quiz will be considered incorrect (Fig. 2d) and options for resuming will be offered. After finalizing all cases at a level, the results screen will display the number of correct and wrong questions. Students at Level 1 will be shown whether they are ready to move to the next level (this occurs if the correct answers represent 70% or more of the questions). Level 1 can be resumed if the required score is not achieved.

In Fig. 2e, the student will access the panel page, where the progress will be shown (number of correct questionnaires in percentage), the time spent answering all questions, and the number of questions answered correctly at each level. On this page, the student can also start the questionnaire for any of the levels (to start the questionnaire from Level 2, he must have answered correctly at least 70% of the Level 1 questionnaires) by clicking on the “Redo” button.

Finally, in Fig. 2f, which is the classification page, the student will be shown his general classification in relation to all other students. Below, there will be a bar showing the number and percentage of correct answers for each level. For the ranking calculation, the number of correct answers is considered as well as the number of errors and the response time at each level.

In addition, the student will have access to supplementary material that will serve as a theoretical complement to support the use of the system. This material can be accessed in the main page or in appropriate sections at the end of each quiz.

## Discussion

The SmartPath^k^ program was developed using supervised machine learning, which proved to be reliable when inferring the diagnosis of glomerular diseases in the absence of the nephropathologist, thereby aiding in the teaching of this discipline through a complementary mode of learning on a virtual platform. This is highly relevant in the current situation of the new coronavirus pandemic, which has triggered significant changes in the ways of teaching and learning, as digital educational tools are the only possible current alternatives, as well as in post-pandemic education.

Digital teaching requires computer knowledge of the pathologist or teacher so that learning is as efficient as possible. To address the need for computer training in pathology residency programs, three organizations—the Association of Pathology Chairs (APC), the Association for Pathology Informatics (API), and the College of American Pathologists (CAP)—convened a working group composed of computer specialists and authorities in medical education in graduate courses in pathology. This working group, led by a core team of IT and education leaders from the three organizations, developed and launched the “Pathology Informatics Essentials for Residents” (PIER), a flexible curriculum project to educate computer pathology residents [23].

In addition to this project, several other methodologies, mainly using AI algorithms, are being validated for use in the routine work of the nephropathologist’s laboratory aiming at better accuracy and speed in the assessment of punctual morphological findings of renal biopsies [9, 21, 24]; however, there has as yet been no use of such methods in routine laboratory work or for educational support, such as the system developed in this study.

SmartPath^k^ is an intelligent, dynamic, and open system that allows the insertion of a new case as soon as it is available, thus generating a new decision tree and accuracy to provide students with new experiences with each new quiz generated and analyzed in the SmartPath^k^ tool.

Learning takes place dynamically through student interactions with SmartPath^k^ via quizzes, image analysis, and access to the supplementary material. In addition, the student is placed in a class under the guidance of a teacher who when necessary can be consulted. The teacher also has access to the performance evaluation of the class, allowing the detection of weaknesses in content and reinforcement to improve learning.

The use of quizzes allows instructors to deepen, consolidate, reinforce, and evaluate student learning. Its main objective is to encourage students to think, research, reflect on, and discuss the contents of the classroom through theoretical and practical questions. Therefore, we believe that the use of quizzes in SmartPath^k^ developed through its intelligent and dynamic construction of questions can contribute to the teaching / learning process based on Information and Communication Technologies. We will soon validate in a real face-to-face classroom setting whether the tool offers a satisfactory performance as a complementary digital device in the teaching-learning process of a group of graduating nephropathology specialists. Because of the pandemic, there was not any group with the previous level of knowledge required for the use and validation of the tool, what is to be accomplished in a future phase of the study.

## Conclusions

SmartPath^k^ is the first program developed to support teaching in nephropathology using AM for the diagnosis of glomerulopathies, with the purpose of increasing the educational training capacity of new medical professionals in renal pathology. It is dynamic and intelligent, as it allows the inclusion of new cases without the need to modify the program by Computer Science professionals.

The use of the SmartPath^K^ system entails a low operational cost because only devices connected to the internet are needed for its use. SmartPath^K^ can be used at the following address: http://smartpathk.pmadt.com.br/. From this address, it is possible to access the system with a test account (login: test; password: test) and evaluate the system using data that are already available.

## Supplementary Information

**Additional file 1: Table 1.** List of Sample Glomerulopathies.

## Data Availability

Project name: SmartPath^K^. Project home page: http://smartpathk.pmadt.com.br/ Operating system(s): Platform independent. Programming language: PYTHON. Other requirements: not applicable. License: proprietary software. Any restrictions to use by non-academics: license needed.

## Abbreviations

ML: Machine learning
APC: Association of Pathology Chairs
API: Association for Pathology Informatics
CAP: College of American Pathologists
AI: Artificial Intelligence
PIER: Pathology Informatics Essentials for Residents
