# Supplementary material for: Smartpathk: a platform for teaching glomerulopathies using machine learning
Source: BMC Med Educ. 2021 Apr 29;21:248. doi: 10.1186/s12909-021-02680-1 (PMC8084264; doi:10.1186/s12909-021-02680-1)
Supplement: Supplementary file 1 — Additional file 1: Table 1. List of Sample Glomerulopathies. [file 12909_2021_2680_MOESM1_ESM.pdf]

Table 1: List of Sample Glomerulopathies

Minimal Change Disease
Normal Kidney
Focal and Segmentar Glomerulosclerosis
Primary Membranous Glomerulopathy
Secondary Membranous Glomerulopathy
Thin Membrane Disease
Iga Nephropathy (Berger's Disease)
Membranoproliferative Glomerulonephritis Type I
Membranoproliferative Glomerulonephritis Type II (Dense Deposit Disease)
Membranoproliferative Glomerulonephritis Type III
Acute Diffuse Glomerulonephritis (Gnda)
Diabetic Nephropathy
Hypertensive Vascular Nephrosclerosis
Focal And Global Lupic Glomerulonephritis - Class III
Diffuse And Segmental Lupic Glomerulonephritis Classe IV
Podocytopathies (Minimal Lesions Vs. Focal Segmental And Focal Glomerulosclerosis)
Membranous Lupic Glomerulonephritis - Class V
Fabry Disease
Podocytopathy: Unsampled Segmental And Focal Glomerulosclerosis
Diffused And Global Lupic Glomerulonephritis - Class IV
Proliferative Mesangial Lupic Glomerulonephritis - Class II
Segmental And Focal Lupic Glomerulonephritis - Class III And Membranous Association (Class V)
Crescent Glomerulonephritis
Membranoproliferative Glomerulonephritis
Segmental And Focal Lupic Glomerulonephritis - Class III
Membranous Glomerulopathy
Segmental And Focal Glomerulosclerosis Collapsing Variant
Amyloidosis
Fibronectin Glomerulopathy
Thrombotic Microangiopathy
